# Cells in ExperimentaL Life Sciences (CELLS-2018): capturing the knowledge of normal and diseased cells with ontologies

**DOI:** 10.1186/s12859-019-2721-9

**Published:** 2019-04-25

**Authors:** Sirarat Sarntivijai, Yongqun He, Alexander D. Diehl

**Affiliations:** 1 ELIXIR, Wellcome Genome Campus, Hinxton, Cambridge, UK; 2 University of Michigan Medical School, Ann Arbor, MI USA; 3 Department of Biomedical Informatics, Jacobs School of Medicine and Biomedical Sciences, University at Buffalo, Buffalo, NY USA

## Abstract

Cell cultures and cell lines are widely used in life science experiments. In conjunction with the 2018 International Conference on Biomedical Ontology (ICBO-2018), the 2nd International Workshop on Cells in ExperimentaL Life Science (CELLS-2018) focused on two themes of knowledge representation, for newly-discovered cell types and for cells in disease states. This workshop included five oral presentations and a general discussion session. Two new ontologies, including the Cancer Cell Ontology (CCL) and the Ontology for Stem Cell Investigations (OSCI), were reported in the workshop. In another representation, the Cell Line Ontology (CLO) framework was applied and extended to represent cell line cells used in China and their Chinese representation. Other presentations included a report on the application of ontologies to cross-compare cell types and marker patterns used in flow cytometry studies, and a presentation on new experimental findings about novel cell types based on single cell RNA sequencing assay and their corresponding ontological representation. The general discussion session focused on the ontology design patterns in representing newly-discovered cell types and cells in disease states.

## Introduction

The rapid advancement of cell technologies has inevitably led to challenges in keeping up with the volume of the data being produced as well as the dynamic evolution of data formats and knowledge representation. Experimental cell cultures and cell lines are widely used and often generated de novo as part of an experimental protocol, while normalization of experimental cell data produced in different laboratory settings is difficult due to the non-synchronous nature of multiple laboratories working on similar questions on the same timeline. New knowledge obtained by high-resolution technologies (e.g., mass cytometry or single-cell RNA sequencing) adds more data volume that requires robust analysis and representation, especially regarding novel cell populations that do not correspond to existing classes of the community-based Cell Ontology (CL) [1] or Cell Line Ontology (CLO) [2], two cell-related reference ontologies in the Open Biomedical Ontology (OBO) framework [3].

The first International Workshop on Cells in ExperimentaL Life Science (CELLS-2017) was held in Newcastle, UK, on September 2017 on the theme of identifying challenges from the ontology semantic perspective, and the experimental laboratory perspective [4]. The CELLS-2017 workshop included 7 oral presentations, and eventually five full-length articles [5–9] were published in the special issue of the first CELLS workshop in *BMC Bioinformatics* (https://bmcbioinformatics.biomedcentral.com/articles/supplements/volume-18-supplement-17).

Following the success of the first CELLS workshop, the 2nd International Workshop on Cells in ExperimentaL Life Science (CELLS-2018) was held on August 7, 2018, in conjunction with International Conference on Biomedical Ontology 2018 (ICBO-2018), August 7–10, 2018, Corvallis, Oregon, USA. CELLS-2018 was organized with a focus on the themes of addressing the challenges of knowledge representation of newly-discovered cell types and of cells in disease states.

## Summary of the talks and papers presented at this workshop

The half-day CELLS-2018 workshop included five oral presentations and a general discussion session. Four of the proceedings papers, corresponding to four oral presentations, have been extended, further reviewed, and are published in this special issue for the CELLS-2018 workshop in *BMC Bioinformatics*.

In this workshop, two new ontologies were reported. Serra et al. presented the development of the Cancer Cell Ontology (CCL) that represents cancer cell types related to a variety of hematologic malignancies using logical axioms that capture the expression of cellular surface markers using Protein Ontology (PRO) [10] classes [11]. The ontology includes human and computer-readable definitions for 300 classes of blood cancers. The logical axioms of the ontology can be used to classify patient cell surface marker data into appropriate diagnostic categories, to allow for integration of the ontology into existing tools for flow cytometry data analysis to facilitate the automated diagnosis of hematologic malignancies.

The other reported new ontology is the Ontology for Stem Cell Investigations (OSCI) [12]. Stem cells and stem cell lines have been widely used in biomedical research. CL and CLO represent in vivo stem cells and in vitro stem cell line cells, respectively. However, neither CL nor CLO has a focus on the representation of stem cell investigations, which is the main goal of the community-driven OSCI ontology. OSCI imports stem cell and stem cell line related classes from CL and CLO. OSCI also imports classes needed to describe stem cells and stem cell experimental methods from other ontologies such as PRO [10] and the Ontology for Biomedical Investigations (OBI) [13] . Many metadata types associated with various stem cell investigations have been identified and represented as classes in OSCI. Two use cases of OSCI include applying OSCI to systematically explore experimental variables in induced pluripotent stem cell line cell studies related to bipolar disorder, and applying OSCI to model and represent stem cell gene markers and relations identified using semi-automated literature mining.

The Chinese National Infrastructure of Cell Line Resource (NICR) stores and distributes over 2000 cell lines in China. To support worldwide interoperable cell line information representation and usage, Pan et al. reported the development of CLO-NICR ontology that represents these cell lines using the semantic framework of CLO [14]. As a subset of the master CLO release, CLO-NICR can also be used in a stand-alone way to support applications for biomedical research in China NICR. A new ontology design pattern specifically to CLO-NICR is proposed to bridge cell line cells to disease studies using laboratory animal models. The authors also demonstrate the Chinese language representation of the cell line cell information. The CLO-NICR development was one of the research efforts from the OntoChina (http://www.ontochina.org) [15], a new initiative supporting an ontology developer community program in China to promote collaborative ontology development and applications around the world.

In flow cytometry, cells are gated into different populations based on common features like forward or side scatter or expression patterns of protein markers. The cell populations are typically identified via a general name of the cell type (e.g. ‘T cells’) and the specific marker pattern observed (e.g. CD14−/CD3+). In a CELLS-2018 presentation, Vita et al. applied ontologies to connect cell population descriptions and gating definitions [16]. In their study, ontologies were used to cross-compare cell types and marker patterns in the ImmPort Immunology Database and Analysis Portal [17]. The gating definitions were parsed using classes from the Cell Ontology (CL) [1] and the Protein Ontology (PRO) [10]. Logical axioms in the CL were used to detect discrepancies between submitted data about cell populations and cell type classes in the CL. New OBI classes were also generated to describe and capture data acquisition. This study resulted in suggestions for new logical axioms and cell types for addition to the CL.

One challenge is how to ontologically represent the cell types newly discovered using experimental technologies. In a CELLS-2018 podium presentation, Mohamed Keshk of Richard H. Scheuermann’s group presented a provisional cell type ontology that represents newly identified novel cell types experimentally identified using single cell RNA sequencing (scRNAseq), an extension of the work Dr. Scheuermann’s group presented in the CELLS-2017 workshop [5]. The data being gathered from the experimental work is represented in a standard semantic format that can be exchanged, retrieved, and inferred over using standard approaches and tools. This format supports the translation and representation of biological knowledge to become findable, accessible, interoperable and reproducible (FAIR), per the FAIR principles [18].

As can be seen, the presentations at the CELLS-2018 workshop covered a range of issues in the representation of cell types: cell types and experimental methodology related to them from under-represented domains including cancer cells and stem cells, the ontological representation of cell lines from a major national resource in China, and the challenge of representing experimental results about cells based on flow cytometry or single cell RNA sequencing. If there is a common theme to these efforts, it is the drive to present a coherent ontological representation of existing and new knowledge about cells that builds upon existing efforts in the CL, CLO, and OBI, and is likewise extensible for the representation of new data and methodology related to cell types, within the OBO Foundry framework.

## Challenges and opportunities

In the CELLS-2018 workshop, we also organized a general discussion session. Our discussion focused on the design pattern representation of normal vs diseased conditions in cells. For example, what is the difference between normal and diseased cells, and how to ontologically represent the transformation from a normal cell to diseased cell, and what are the common design patterns for normal and diseased cells? In addition, we discussed how to represent additional information (e.g., gene markers) in ontology? The five presentations described above fit in well with the discussion theme.

One challenge in cell ontology development is to how to specify the definition of cell types. The de facto convention used for the creation of many classes in the CL has resulted in the creation of cell type classes that are named and defined both textually and logically by the cell surface markers expressed by the cell type using a *post-composed* approach. This is may be less than ideal as the community discovers new cell types based on subdividing known cell populations with additional markers. As we move beyond marker definition of cell types developed via flow cytometry to defining cell types via single cell RNA sequencing or proteomics, a cell type could potentially be defined by a combinations of hundreds of marker genes or proteins. Should we add more markers to cell types and use these markers for cell type definitions? As reflected by the work of Dr. Scheurmann’s group and others [19], we might therefore consider what constitutes the minimal combination set of markers that can uniquely define a new cell type? Under some circumstances, defining cell types by cell surface or internal markers may not be sufficient. For example, to define brain cells, we may also need to include other physiological and morphological properties such as cell location and function in order to uniquely define a specific brain cell type. However, in many older experimental protocols, all the cells of a given tissue are homogenized prior to RNA or protein extraction, resulting in the loss of specific location and function information in the process. Other types of experiment rely on, for instance, immunofluorescence or green fluorescence protein (GFP) tagging to examine the expression and localization of protein markers in cells. Given the diversity of the experimental approaches for studying cells, we need to be careful in how we represent experimental results in the form of ontological definitions of cell types. Some results may be true under specific experimental conditions, such as during cellular activation; however, under other conditions, the results may not be true or may only be true for a particular state of a cell type. Ontology classes should always represent certainty, and not probability. Definitive identification and representation of cell types requires synthesizing knowledge gained from multiple experimental approaches for a cell type definition to be accepted as truth. Another question is how to infer cell function. How can we ontologically represent cell function and related pathways in normal or diseased cells? Can we infer the function by merging cell morphology and gene expression? Can we use function analysis via, for instance GO term enrichment of expressed genes to identify key biological processes a cell may be participating in, and thereby establish a functional signature that can be used to infer cell type? These questions may need to be addressed outside an ontology class level. Solutions under discussion include inference via instances of the main class, or using other object/annotation properties to build the inferred knowledge.

As the basic unit of life, the cell remains central to biological and biomedical research. Given that challenges presented by studies of cells and their ontological representation and applications, the attendees of the workshop welcome and expect more CELLS workshops in the future. And, of course, these challenges in themselves are also opportunities. For example, the Human Cell Atlas project, which aims to map all the cells in the human body, has recently been initiated from vision to reality [20], and is already generating a wealth of data about cell types. Many precision medicine studies, such as the Kidney Precision Medicine Project (KPMP: http://kpmp.org), aim to understand specific cell-disease associations and guide the control and treatment of diseases under different clinical conditions [21]. A variety of recent studies have discovered many new cell types, which sometimes show different characteristics in normal or disease states [22–26]. These clinical, experimental, and disease conditions, as well as new knowledge about cell types, need more in-depth ontological modeling, representation, and analysis. New tools are also needed to support more productive ontological research in the cell field, including the development of machine learning methods that work across many data sets to identify necessary and sufficient criteria for cell type definitions across the whole body. We anticipate promising research progress in the cell-related ontology field in the years to come.

## Conclusion

The CELLS-2018 workshop provided a venue for researchers to report their updates and new studies, and to discuss challenges and innovative solutions in the development and application of biomedical ontologies to represent and analyze in vivo and in vitro cell- and cell line-related knowledge and data, including stem cell technologies.
